# Weekday time in bed and obesity risk in adolescence

**DOI:** 10.1002/osp4.455

**Published:** 2020-09-21

**Authors:** Yngvild Sørebø Danielsen, Ståle Pallesen, Børge Sivertsen, Kjell Morten Stormark, Mari Hysing

**Affiliations:** ^1^ Department of Clinical Psychology University of Bergen Bergen Norway; ^2^ Department of Psychosocial Science University of Bergen Bergen Norway; ^3^ Norwegian Competence Center for Sleep Disorders Bergen Norway; ^4^ Department of Health Promotion Norwegian Institute of Public Health Bergen Norway; ^5^ Department of Research & Innovation Helse Fonna HF Haugesund Norway; ^6^ Department of Mental Health Norwegian University of Science and Technology Trondheim Norway; ^7^ Regional Centre for Child and Youth Mental Health and Child Welfare NORCE Norwegian Research Centre Bergen Norway; ^8^ Department of Health Promotion and Development University of Bergen Bergen Norway

**Keywords:** adolescent, BMI, longitudinal, obesity, sleep duration

## Abstract

**Introduction:**

Sleep curtailment is associated with obesity in children, but few studies have investigated this relationship in a longitudinal sample of adolescents. The aim of the present study was to examine the longitudinal association between weekday time in bed (TIB) at age 10–13 and overweight at age 16–19.

**Methods:**

Adolescents and their parents (*N* = 3025 families), participating in a longitudinal population‐based study, completed questionnaires assessing habitual bedtime and wake time on weekdays, weight and height, socioeconomic status (SES), internalizing mental health problems and disturbed eating. Two surveys were administered with a 6‐year interval (T1 and T2). A one‐way analysis of covariance (ANCOVA) was performed examining the association between TIB and weight category 6 years later, with SES, internalizing problems and disturbed eating at baseline entered as covariates. Hierarchical and logistic regression analyses were used to assess TIB at age 10–13 years to as a predictor of body mass index (BMI) standardized deviation scores (SDS) and overweight status at age 16–19 adjusting for the same confounders and baseline BMI.

**Results:**

A linear inverse relationship between TIB at age 10–13 and BMI category at age 16–19 was demonstrated by the ANCOVA, *p* < 0.001. Shorter TIB was related to higher weight, but the effect size was small (partial eta squared = 0.01). When adjusting for the included baseline confounders in the hierarchical regression model TIB significantly predicted later BMI SDS (*β* = −0.039, *p* = 0.02). The adjusted logistic regression model showed that for each hour reduction of TIB at T1 the odds of being overweight/obese at T2 increased with a factor of 1.6.

**Conclusion:**

Shorter TIB was found to be a significant, yet modest, independent predictor of later weight gain in adolescence. The findings implicate that establishing healthy sleep habits should be addressed in prevention and treatment strategies for adolescent obesity.

## 1 INTRODUCTION

Sleep duration has been markedly reduced parallel to weight gain in children and adolescents over the last decades,^1^, ^2^, ^3^ and short sleep duration constitutes an independent and modifiable risk factor for obesity among children and adolescents.^4^, ^5^, ^6^ Studies have reported cross‐sectional and longitudinal associations between short sleep duration and obesity.^7^, ^8^, ^9^, ^10^, ^11^, ^12^, ^13^, ^14^, ^15^ The causality of this relationship is still debated, as well as the underlying mechanisms.^16^, ^17^ Proposed pathways for short sleep to instigate weight gain include changes in appetite regulating hormones (e.g., leptin and ghrelin), higher energy intake, changes in food preferences and eating patterns, negative emotions leading to emotional eating, less energy expenditure due to more sedentary time (including screen time), and reduced physical activity as a consequence of high levels of fatigue and sleepiness due to sleep loss.^16^, ^18^, ^19^ A comprehensive review from 2019, addressing these different pathways, found inconclusive evidence for changes in leptin and ghrelin due to shorter time in bed in children and adolescents.^19^ While higher calorie intake was demonstrated across all experimental studies restricting time in bed, there were no associations in cross‐sectional studies.^19^ However, poorer dietary quality was related to shorter weekday time in bed (TIB) in most studies. The review further reported growing evidence for shorter TIB being related to preferences for sweet, salty, fatty, starchy, or high glycemic foods, more external and emotional eating, skipping breakfast as well as more sedentary time and screen time.^19^ The evidence for a relationship of TIB to moderate and vigorous activity was inconclusive.^19^

Reviews demonstrate that nearly all cross‐sectional studies on children find short sleep duration to be related to obesity, while for adults the findings are less consistent.^7^, ^8^ Longitudinal studies on the association between short sleep duration and subsequent weight gain likewise report a consistent relationship in children, but inconsistent results for adults.^9^ A meta‐analysis published in 2015 based on 22 longitudinal studies on the association between sleep duration and body mass index (BMI) in children and adolescents concluded that children with short sleep duration had about twice the odds of becoming overweight or obese compared to normal sleeping peers,^13^ and further that adolescents were at higher risk than younger children.^13^ These findings were supported in a meta‐analysis from 2018 concluding that short sleep duration is a significant predictor of the development of overweight and obesity in both middle childhood and adolescence, albeit children were found to be at higher risk than adolescents.^15^ However, only three studies on adolescents were included.^15^

Together these studies indicate that children are more vulnerable to gain weight due to sleep loss than adults. The potential mechanisms underlying this difference is not well established. However, the finding may reflect that the effect of short sleep on weight gain is strongest for the period immediately following the transition to short sleep.^9^ For longitudinal studies this would implicate that the period between the measurements should include the phase where transitions to shorter sleep duration occurs in order to be able to demonstrate a relationship to weight change. Several of the longitudinal studies from adolescent populations include a time span of only 1 year, which might be too short to be sensitive to detect a potential relationship between short sleep and weight gain.^20^

Sleep duration has been significantly reduced in children, and even more in adolescents, over at least the last 30 years.^1^ Lifestyle habits related to food, electronic media use, and physical activity have also changed among adolescents in the same time period.^21^, ^22^, ^23^, ^24^ Electronic media devices like smartphones and tablets are now often present in the bedroom at night and used for engaging in social media platforms, watching TV‐shows and gaming.^23^ Furthermore, a review on changes in adolescent diet found, increased snacking, more eating away from home, higher intake of fast food, processed food, and sweetened beverages,^24^ even though trends are somewhat disparate in different countries.^25^ Thus, adolescence might be a particularly vulnerable period for short sleep to instigate weight gain.

In terms of research methodology, the importance of adjusting for possible relevant confounders has been highlighted when examining the prospective relationship between sleep duration and weight changes.^20^ A large study among the US adolescents demonstrated for example that the short sleep–obesity association was no longer significant when adjusting for confounders such as depression and duration of television viewing.^26^ Notably, depression is associated with the development of obesity in adolescence.^27^, ^28^ Furthermore, sleep problems are also symptoms of depression^29^ and depression is highlighted as one of the most important factors to adjust for in studies examining the link between sleep and obesity in adolescence.^20^ It may both be a confounder, but also a mechanism in the causal pathway between short sleep duration and obesity. Furthermore, disturbed eating has been found to be an important predictor for weight gain and the development of obesity during adolescence^28^, ^30^ and thus relevant to include as a covariate when investigating the longitudinal sleep–weight association. Socioeconomic status (SES) has likewise demonstrated associations to both sleep habits and obesity.^28^, ^31^, ^32^ Only a few studies have examined prospective associations between sleep duration and weight status in adolescence, and the findings remain inconclusive.^13^, ^14^, ^15^, ^20^ The present study adds to the literature by using a 6‐year time frame between measurements in a population‐based sample during a critical phase of development.

The aim of the present study was to examine the association between TIB at age 10–13 and weight status at age 16–19, adjusting for baseline BMI standardized deviation scores (SDS) and relevant psychosocial factors (SES, disturbed eating patterns, and internalizing problems). Both the association between TIB and general weight gain (BMI SDS) over this 6‐year period, and the ability of TIB to predict later overweight status were examined.

## 2 MATERIALS AND METHODS

### 2.1 Design and sample

Data stem from the second (T1 in the current study) and forth (also labeled “the youth@hordaland survey”; T2 in the current study) wave of the Bergen Child Study (BCS). The BCS is a longitudinal total population study of three cohorts of children. The main aim of the BCS was to assess the prevalence of mental health problems in children and the availability and use of health care services. All primary school children born in 1993–1995 (*N* = 9430) in the Bergen municipality area were invited to participate. The data analyzed in the present study included a longitudinal sample of participants from T1 (age 10–13 years) and T2 (age 16–19 years) of the BCS (*N* = 3025). Data from participants with both valid parent‐reported assessment at T1 (age 10–13) and self‐report at T2 (age 16–19) were included in our sample.

A total of 5781 children/parents participated at T1 during spring of 2006 (T1). Information about the study and questionnaires were distributed to all children in the 5th and 6th grade (*N* = 9430) of all schools in the Bergen municipality area, and the schools further collected the questionnaires from the families that agreed to participate. At T2, all adolescents attending secondary education in the Hordaland county, in which Bergen is the largest city, were invited during spring 2012 (T2). A total of 10,254 of the 19,439 invited adolescents participated (participation rate of 53%). Information about the study was administered via their official school e‐mail address, and 1 hour during school time was allocated for completion of the survey. Those not in school received information by postal mail to their home address. The survey was web‐based and included questions about different mental health issues, physical activity, sleep, self‐reported height and weight, and health care use.

### 2.2 Measures and procedure

#### 2.2.1 Body mass index

At the first measurement (T1), BMI (kg/m^2^) was calculated based on the child's parent‐reported height and weight. Age‐ and gender‐adjusted BMI SDS was computed using the reference from the International Task Force for Obesity (IOTF) with corresponding cutoffs for underweight, normal weight, overweight, and obesity.^33^ At the second measurement (T2), BMI was based on the adolescents' self‐reported height and weight. IOTF cutoffs for normal weight, overweight, and obesity were used for those below 18 years.^33^ For the participants above 18 years, the weight class categorizations from the World Health Organization were used.^34^ Overweight and obesity were categorized together, and normal weight and underweight constituted the other group in the logistic regression analysis.

#### 2.2.2 Weekday time in bed

At T1, parents reported habitual bedtime for their children at weekdays as well as habitual wake time. TIB was calculated based on the reported bedtimes and rise times. Furthermore, TIB was operationalized both as a continuous variable and a categorical variable. Categorization was made based on recommendations for adequate sleep duration at different ages from the National Sleep Foundation (shorter than recommended, within recommended sleep duration, and longer than recommended sleep duration).^35^ According to these guidelines, recommended sleep duration for 6–13 year olds is 9–11 h, for 14–17 year olds 8–10 h, and for 18–25 year olds 7–9 h,^35^ respectively.

#### 2.2.3 Internalizing problems

The Strength and Difficulties Questionnaire (SDQ) is a screening tool for mental health problems in children and youths.^36^, ^37^ The scale consists of 25 items constituting five subscales: Emotional Symptoms, Conduct Problems, Hyperactivity, Peer Problems, and Pro‐Social Behavior. The response categories for each item is: “*Not true*,” “*somewhat true*,” and “*certainly true.*” Subscale scores range from 0 to 10. The reliability and validity of the SDQ and the psychometric properties are generally found to be satisfactory.^38^, ^39^, ^40^ Parent reporting at T1 was used for the purpose of the present study and the scales for emotional symptoms and peer problems were combined to represent a measure of internalizing problems.^41^ The Cronbach's alpha for the SDQ emotional subscale in the BCS sample was 0.65.

#### 2.2.4 Disturbed eating

The Eating Disturbance Scale (EDS‐5)^42^ is a self‐report measure consisting of five questions related to eating disorder symptomatology. Item scores range from 0 to 3 (0 indicating “no problems” and 3 “high degree of problems”). A sensitivity and specificity of 0.90 and 0.88 for the EDS‐5 with respect to DSM‐IV eating disorders have previously been reported.^42^ Data from the parent report version at T1 were used for the present study, for which the Cronbach's alpha was 0.73.

#### 2.2.5 Socioeconomic status

The educational level of the mothers and fathers were reported as one of five different categories: *“Secondary school”, “vocational high school”, “high school”, “university/college 4 years or less”, or “university/college more than 4 years”* at T1 (0‐5). Perceived family financial circumstances were evaluated on a five‐point scale ranging from “very good” to “very poor”. This measure has been found to correlate reasonably well with objective measures of income in a subsample of participants.^43^ A composite score including the education level of both parents and perceived family financial circumstances was used as a proxy for SES in this study (the scores on the three subcategories were added together constituting a measure ranging from 0 to 15).

### 2.3 Statistical analyses

IBM SPSS Statistics 24 (SPSS Inc.) was used for all analyses. Means and standard deviations for the dependent and independent variables were calculated as well as how scores on BMI categories were distributed dependent on sleep duration. A McNemars test was conducted to examine whether the proportions of individuals belonging to the different weight categories were different at T1 and T2.

A one‐way analysis of covariance (ANCOVA) was performed with weight status at T2 as independent variable and TIB at T1 as dependent variable. SES, EDS‐5 disturbed eating, and SDQ internalizing problems were entered as covariates. Estimated marginal means (EMM) for TIB at T1 with 95% confidence intervals were computed stratified by weight status at T2, adjusting for SES, disturbed eating and internalizing problems at T1.

Preliminary analyses were conducted to ensure no violation of the assumptions of normality, linearity, and multicollinearity.

Hierarchical multiple regression analysis was used to assess the ability of TIB at age 10–13 years to predict BMI SDS scores at age 16–19. Step two of the analyses adjusted for baseline confounders (BMI SDS, SES, EDS‐5 disturbed eating, and SDQ internalizing problems). A model including TIB as quadratic variable was also investigated to examine whether the association between sleep and BMI SDS was curvilinear or linear in the current sample.

Logistic regression analysis was performed to assess the impact of TIB at age 10–13 on the likelihood that the adolescents reported BMI in the range of overweight/obesity compared to normal weight/underweight at age 16–19. Step two of the analysis adjusted for baseline confounders (BMI SDS, SES, EDS‐5 disturbed eating, and SDQ internalizing problems).

Both waves were approved by the Regional Committee for Medical and Health Research Ethics in Western Norway (REC‐numbers: REK 062‐06 (T1) and REK 2011/811 (T2)). For the first time point written‐informed consent was obtained from all parents included in this study. For youth@hordaland, parents were informed about the study while the adolescents themselves consented to participate, as Norwegian regulations state that individuals aged 16 years and older are required to consent themselves.

## 3 RESULTS

### 3.1 Demographical and clinical characteristics of the sample

In all, 3025 adolescents participated at both T1 and T2. Valid BMI scores were obtained for 2525 adolescents at T1 and 2934 at T2, respectively. The sample with valid BMI scores consisted of 55.6% girls and 44.4% boys. Mean age at T1 was 11.7 (range = 10.4–13.4) and 17.4 (range = 16.1–19.3) at T2. In all, 56.3% of the mothers and 55.3% of the fathers had a university/college education. Perceived financial circumstances were evaluated as very good by 17.2%, good by 54.5%, average by 26.2%, poor by 1.8%, and very poor by 0.3% of the total sample, respectively. The sample with valid BMI scores did not differ significantly from the total longitudinal sample on gender, age, BMI, BMI SDS, time in bed, internalizing problems, or disturbed eating. The group without BMI data did, however, report significantly lower SES (composite score described over) (*M* = 10.6, SD = 2.6) compared to those reporting BMI data (*M* = 10.8, SD = 2.5; *t* (2766) = −2.1, *p* = 0.03). Baseline characteristics of the total longitudinal sample are presented in Table 1.

**TABLE 1:** Baseline characteristics of the total longitudinal sample (*N* = 3025)

	*N*	Min	Max	Mean/%	SD
Gender (% girls)	3025	‐	‐	54.9 %	
Age	3025	10.42	13.41	11.74	0.83
Parent‐reported TIB (hours)	2972	7.00	13.50	9.80	0.56
BMI	2525	11.72	52.67	17.95	2.70
BMI SDS	2525	4.90	3.94	0.17	1.07
SES (parent education and economy, 0–15)	2768	4.00	15.00	10.79	2.49
SDQ internalizing problems	3012	0.00	19.00	2.03	2.63
EDS‐5 (disturbed eating)	2988	0.00	7.00	0.27	0.68

Abbreviations: BMI, body mass index; BMI SDS, BMI standard deviation scores based on international task force for obesity references; EDS‐5, Eating Disturbance Scale; SDQ, Strengths and Difficulties Questionnaire; SES, socio economic status; TIB, weekday time in bed.

At T1, IOTF weight categorizations were as follows: 12.4% (*N* = 315) were underweight, 77.6% (*N* = 1959) had normal weight, 8.8% (*N* = 222) had overweight, and 1.1% (*N* = 29) had obesity. At T2 8.2% (*N* = 241) had underweight, 77.6% (*N* = 2277) had normal weight, 11.8% (*N* = 347) had overweight, and 2.4% had (*N* = 69) obesity. A McNemar's test showed that the proportions of individuals belonging to the different weight categories were different at T2 compared to T1, *p* < 0.001. According to parent report at T1 93.0% of the adolescents had habitual week day sleep duration within the recommendations of the National Sleep Foundation at baseline, 4.5% (*N* = 135) were categorized as short sleepers, and 2.4% (*N* = 73) as long sleepers for their age.^35^ Mean TIB at T1 was 9:48 h and at T2 7:28 h. Baseline weight categorization of the sample stratified by sleep duration is presented in Table 2.

**TABLE 2:** Baseline weight categorization of the sample stratified by sleep duration

	Short sleepers (*N* = 157)	Normal sleepers (*N* = 2167)	Long sleepers (*N* = 74)
Underweight	16 (10.2 %)	264 (12.2 %)	11 (14.9 %)
Normal weight	123 (78.3 %)	1685 (77.8 %)	58 (78.4 %)
Overweight/obesity	18 (10.5 %)	218 (10 %)	5 (6.7 %)

*Note*: Weight categories are determined by criteria from International Task Force for Obesity; categorization of sleep duration is based on recommendations from National Sleep Foundation.

There was no significant mean difference between TIB in the group of adolescents with overweight/obesity and the group of normal weight peers at T1.

### 3.2 Longitudinal associations between sleep duration and BMI

Preliminary analyses tested the interaction effect between gender and the main predictor variable on the dependent variables for both the ANCOVA, and hierarchical and logistic regression analyses. No significant interaction effects involving gender were found, all *p's* > 0.287. Thus, gender was not included as a covariate in the analyses, and separate analyses for boys and girls were not conducted. The number of participants with overweight were also considered too small to conduct stratified analyses for boys and girls.

**FIGURE 1:**
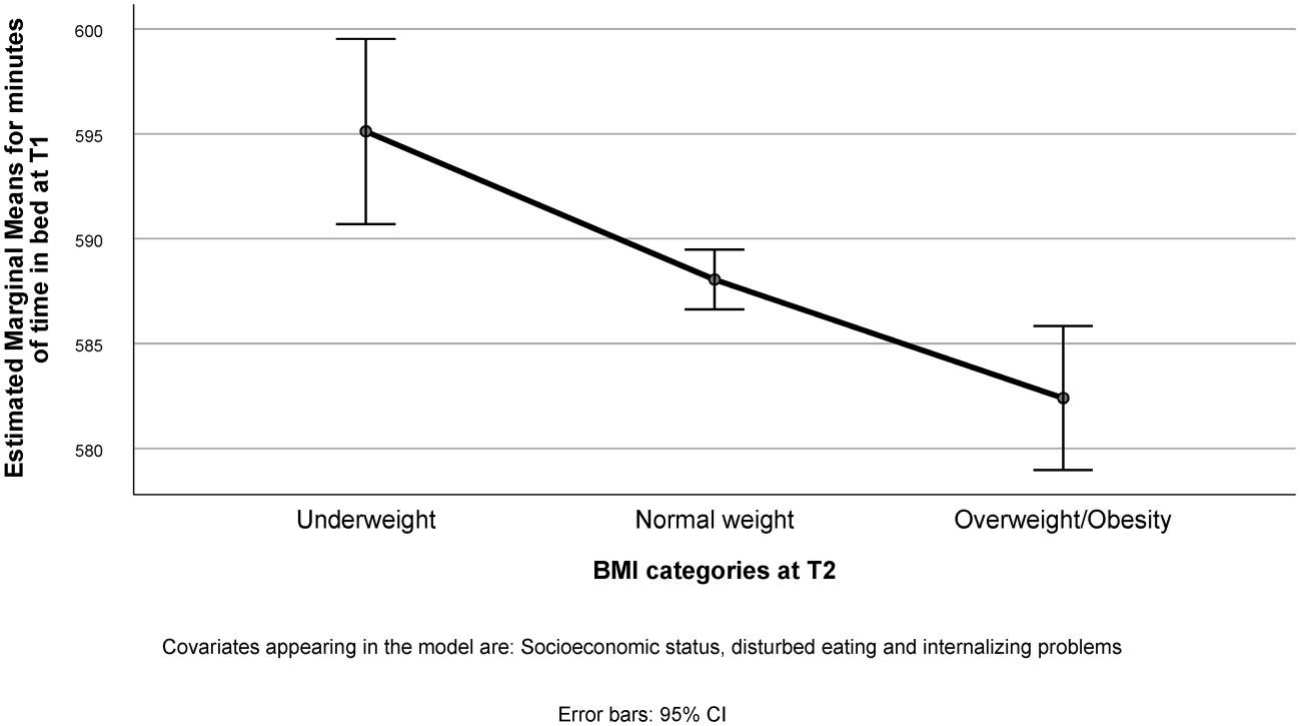
Estimated marginal means for minutes of weekday time in bed at T1 and weight category at T2. BMI, body mass index

Figure 1 displays the EMM for TIB at T1 by BMI category at T2. After adjusting for all covariates, overweight/obese adolescents at T2 spend significantly shorter TIB (9:42 h) at T1 compared to both adolescents with normal weight (9:48 h) and underweight (9:55 h; *p* < 0.001). The effect, however, was small, as indicated by a partial eta squared value of 0.01.

A hierarchical multiple regression analysis was used to assess the ability of TIB at T1 to predict BMI SDS scores at T2 in crude as well as in adjusted analyses. The adjusted analysis controlled for the impact of BMI SDS at T1, internalizing problems, disturbed eating, and SES. In the crude analysis, TIB explained 0.4% of the variance in BMI SDS scores at T2, *p* < 0*.*002. TIB at T1 was significantly and inversely related to BMI SDS score at T2 (*β* = *−*0.06, *p* < 0.002). When adjusting for the included baseline confounders (BMI SDS, SES, disturbed eating EDS‐5, and SDQ internalizing problems) TIB at T1 was still a significant predictor for BMI SDS at T2 (*β* = *−*0.04, *p* = 0.02). A model including TIB as quadratic variable was also investigated but did not contribute significantly in explaining the variance.

A logistic regression analysis was performed to assess the impact of parent‐reported TIB at T1 (hours of time in bed; continuous variable) on the likelihood that the adolescents reported a BMI in the range of overweight/obesity at T2 (overweight/obesity or normal weight/underweight). The crude model was statistically significant, *χ* ^2^ = (1, *N* = 2262) = 12.4, *p* < 0.001, indicating that it was able to distinguish between adolescents having overweight/obesity and normal weight. The odds ratio (OR) for TIB at T1 as a predictor for weight status at T2 was 1.4 (95 % CI = 1.2–1.7). The adjusted model contained five independent baseline variables (parent‐reported TIB, BMI SDS, SES, EDS‐5 disturbed eating, and SDQ internalizing problems). Shorter TIB at T1 still significantly predicted overweight/obesity at T2 in the adjusted model (*beta* = *−*0*.*44, *p* = 0.001.). The OR for TIB at T1 as a predictor for weight status at T2 in the adjusted model was 1.6 (95 % CI = 1.2–2.0). For each hour decline in sleep per night the odds of being overweight/obese increased with a factor of 1.6.

## 4 DISCUSSION

In this study, short TIB at age 10–13 significantly predicted subsequent weight gain and overweight status 6 years later. The association was still significant when adjusting for SES, internalizing problems, and disturbed eating patterns. Short weekday sleep explained a small, but significant, part of the variance in later weight status in adolescence. One hour shorter sleep at age 10–13 was associated with a 1.6 increased odds of having overweight or obesity at age 16–19.

The present findings are in line with results reported from a recent meta‐analysis of prospective studies of short sleep duration as a risk factor for developing overweight and obesity.^15^ The three studies encompassing adolescents combined a risk ratio for short sleepers to develop overweight or obesity of 1.3 (95% CI = 1.1–1.5; *p* < 0.002). Another review from 2012, however, included only two longitudinal studies on sleep duration and weight gain in adolescence, neither of which reported a significant relationship.^20^ One of these studies was a large US study^26^ including 13,568 adolescents. In the adjusted analyses, the authors found that depression and intensive TV‐viewing predicted obesity; however, short sleep duration and parental income were not significant predictors. That study did only have one year interval between measurements, which might be a too short time frame to elucidate the relationship between short sleep and obesity.^9^ Likewise, other studies with 1–2 years intervals between measurements, report no associations between short sleep and weight gain.^44^, ^45^ The majority of studies with a longer time frame, comparable to the present study, do find short sleep to be associated with weight gain.^5^, ^46^, ^47^, ^48^ This suggests that the time frame between measurement of exposure and outcome might be crucial in explaining different findings across studies examining the prospective relationship between short sleep and weight gain.

Which confounders that are adjusted for might likewise explain some of the differences in findings across studies. The adolescent prospective studies of sleep and subsequent weight change, included in relevant meta‐analyses and reviews,^13^, ^14^, ^15^, ^20^ encompassed a variety of different confounders, such as sleep problems, ethnicity, SES, level of physical activity, sedentary activity, eating habits, and depression. Three out of four studies reported no significant prospective association between short sleep and obesity in adolescence when adjusting for depression,^26^, ^44^, ^45^, ^49^ while none of the studies that found a significant relationship adjusted for depression. Depression is thus highlighted as one important confounder to adjust for in future adolescent studies,^20^ hence internalizing problems were included as a confounder in the present study. Contrary to the findings from other studies that included depression as a confounder,^26^, ^44^, ^45^ the present study found that the association between short weekday sleep and weight gain was still significant after adjustment for internalizing problems. There might be several reasons for the discrepancy between the present finding and those from previous studies. First, it could be due to different measures used to assess mental health. The present study included internalizing problems, which comprise both anxiety and depressive symptoms as well as peer problems whereas previous studies adjusted more specifically for depressive symptoms.^26^, ^44^, ^45^ Second, the sample in the present study were slightly younger at baseline than the previous and negative studies.^26^, ^44^, ^45^ Third, it should be noted that the present study had a relatively long follow‐up time (6 years), in contrast to the previous negative studies where the follow‐up time ranged from 1^26^, ^44^ to 2 years.^45^

Findings from epidemiological research might be difficult to translate to clinical practice. Even though the present study, in accordance with others, found shorter sleep duration to be related to obesity risk, also when adjusting for relevant confounders, the effect was small. It is therefore not likely that solely sleeping more would prevent or reduce weight gain markedly. Horne (2011) argues in his review of studies on the association between short sleep and weight gain that only a very small yearly weight gain may be attributed to short sleep.^50^ In the review, it is also pointed to the fact that short sleep in children and adolescents often are linked to a more general lack of family structure and regulation of lifestyle habits and that change of general lifestyle habits may have a greater impact on weight than focusing more narrowly on sleep.^50^ However, including healthy sleep habits as at target, together with other lifestyle habits (e.g., in educational/treatment programs for obesity) would still probably be worthwhile, as sleep habits, screen time, food intake, and physical activity habits all influence each other. In line with this, a review from 2019, discussing intervention studies for obesity addressing sleep, reported some favorable outcomes, including reduced intake of unhealthy snacks and lower overall energy intake.^51^ One study also reported an association between extended sleep duration and reduced BMI following a lifestyle treatment program.^52^

Although utilizing a longitudinal design, including a reasonably large sample and having a long follow‐up time, some limitations of the present study should still be noted. As the prevalence of overweight and obesity were 8.1 % and 1.1 % at T1 in the current sample, group sizes were considered too small to conduct stratified analyses for boys and girls. Furthermore, both weight, height, and sleep duration were self‐ or parent‐reported. Such reports are found to underestimate weight, especially in overweight samples.^53^ However, it is argued that for epidemiological purposes the discriminative validity of parent‐reported weight is sufficient.^54^ Another study from Bergen estimating prevalence levels of overweight and obesity in the same age group using objective measures reported a prevalence of 13.8% of overweight including obesity.^55^ This is approximately the same number as reported in the total baseline sample of the BCS (13%),^56^ indicating somewhat more missing data from overweight than normal weight adolescents in the longitudinal sample used for the current study. An additional limitation is the use of different reporters at the two measurement points. Using TIB as a proxy for sleep duration also reduces the accuracy compared to objective measures that can identify when sleep occurs. Sleep onset latency and wake after sleep onset, which both reduce sleep time, were not accounted for in the definition used in the present study. Furthermore, as only weekday bed time and rise time were used, the present study was precluded from incorporating weekend sleep data, which often are of longer duration than that of weekdays. For some adolescents, a transition to shorter sleep might also occur later than at 10–13 years of age (T1) and not be captured by the measures in this study. Even though the study did adjust for several relevant confounders, it did not include data on other relevant confounders such as sleep quality, physical activity, food intake, or screen time. The attrition from the study could also negatively impact the generalizability of the findings.

## 5 CONCLUSION

Short weekday sleep explains a small, but significant, part of the variance in later weight status in adolescence, also after adjusting for internalizing mental health problems. Sleep habits should, together with other lifestyle habits, be addressed both in terms of prevention and treatment of adolescent obesity.

## CONFLICT OF INTEREST

No conflicts of interest are disclosed.

## AUTHOR CONTRIBUTIONS

Yngvild Sørebø Danielsen was responsible for designing the current substudy, literature review, data analysis, and writing the manuscript. Mari Hysing has participated in planning and data‐collection of the present study and contributed to planning and writing the manuscript. Ståle Pallesen has participated in planning the and writing the manuscript. Børge Sivertsen has participated in planning the manuscript, conducting data analysis, and writing the manuscript. Kjell Morten Stormark has participated in planning the study and data‐collection and writing the manuscript.
